# Intrapartum Results on Differing Degrees of Ketonuria in Nulliparous Women with Gestational Diabetes Mellitus during Spontaneous Labor

**DOI:** 10.1155/2019/7207012

**Published:** 2019-11-11

**Authors:** Shi-Yun Huang, Bo Yu, Xin He, Yi Chen

**Affiliations:** Beijing Obstetrics and Gynecology Hospital, Capital Medical University, Beijing 100026, China

## Abstract

To compare intrapartum results associated with differing degrees of ketonuria in nulliparous women with gestational diabetes mellitus (GDM), we implemented a retrospective cohort study comparing clinical characteristics among differing degrees of ketonuria and the duration and distribution of ketonuria at different stages of labor. We also analyzed adverse maternal and neonatal outcomes for each group. A total of 570 GDM deliveries were included; of these, 238 had negative ketonuria (41.8%), 180 had moderate ketonuria (31.6%), and 152 had ketosis (26.6%). The proportion of patients with a family history of diabetes significantly increased as the degree of ketonuria increased (*P* < 0.001). Moreover, a significantly lower level of HOMA-IR (the insulin resistance index) was observed for the Negative group (*P* < 0.001). The triglyceride (TG) level was significantly higher in the Ketosis group (*P* < 0.001), and the total cholesterol (TC) levels significantly increased as the degree of ketonuria progressed (*P* < 0.001). There were also higher maternal blood sugar levels and a significantly higher proportion of oxytocin augmentation in ketonuria cases (*P* < 0.001). Over three-fourths of patients (75.6%) had a ketonuria duration of ≤2 hours in the Moderate group, 61.2% had a ketonuria duration of between 3 and 4 h in the Ketosis group, and most of the ketonuria cases resolved in the first stage of labor. As the degree of ketonuria progressed, we observed a significantly higher number of cases with fetal heart rate pattern III (FHR pattern III), meconium-stained amniotic fluid III (MSAF III), postpartum hemorrhages, prolonged labor, neonatal hypoglycemia, an umbilical cord arterial pH of <7.2, low Apgar scores, increased neonatal intensive care admissions, augmented forceps-assisted deliveries, and conversions to cesarean sections. The results showed that ketonuria is common during the intrapartum period and that the risk of adverse maternal and neonatal outcomes may increase when complicated with ketonuria.

## 1. Introduction

Gestational diabetes mellitus (GDM), the most common medical complication during pregnancy, is associated with adverse maternal and neonatal outcomes [1]. The incidence of GDM is positively correlated with ketone levels in otherwise healthy pregnant women [2]. Ketone bodies are formed from free fatty acids (FFA), and accelerated lipolysis and increased FFA are responsible for increased ketogenesis during the second half of pregnancy. Moreover, several hormones generated during pregnancy, including progesterone, cortisol, and human placental lactogen, can contribute to a diabetogenic state, presenting as insulin resistance, increased lipolysis, augmented FFA, and ketogenesis [3].

Maternal ketonuria is a hyperosmolar condition, and ketone bodies act as acidic compounds that bind blood bicarbonates and lower serum pH [4]. In 1970, Felig and Lynch first described a type of exaggerated fasting that could result in ketone overproduction during the second trimester of pregnancy [5]. Similarly, Onyeije et al. found that maternal ketonuria among patients with postterm pregnancy was associated with a significant increase in the occurrence of oligohydramnios and a significant deceleration of fetal heart rate [6]. In addition, ketones diffuse freely across the placenta and can be used as a source of energy by the fetus [7], so Kurepa et al. suggested that a mother's hyperketonemia plays a role in fetal development during pregnancy [8]. Previous studies have also shown that maternal ketones elicit potentially detrimental changes in the neurologic statuses of animals and human beings [9]. For example, a ketonic state in diabetic pregnant women has been associated with decreased intelligence and impaired fine motor skills in offspring [10]. Therefore, ketone bodies are believed to induce a variety of maternal metabolic derangements, including dehydration, hyperosmolarity, and ketoacidosis, and to modulate fetal homeostasis [11]. However, many hospital delivery units do not include ketone control among routine recommendations to patients with GDM.

Diabetic pregnant women are predisposed to variations in glucolipid metabolism and may be more prone to ketogenesis compared to their nondiabetic counterparts [12]. Physical stress during labor, compounded by reduced food intake, can also lead to augmented levels of ketones in the blood and urine [13]. The National Institute for Health and Care Excellence (NICE) recommends using variable rate intravenous insulin infusion (VRIII) to maintain capillary glucose levels within a range of 4.0–7.0 mmol/l, as well as monitoring ketonuria during labor. Clear management guidelines for ketonuria have also been recommended in the case of diabetic ketoacidosis (DKA) [14]. However, acute ketoacidosis is an uncommon medical emergency that requires prompt treatment; it is different from ketosis, which is a natural state that allows the body to utilize fat for energy [15]. Although the presence of ketones during pregnancy is considered to be an abnormal physiologic response, it is unknown whether ketones present during labor in normoglycemic women are related to adverse intrapartum results.

Monitoring urinary ketone levels is inexpensive and easily performed by delivery units. However, there are limited data available on ketonuria in patients with GDM during the intrapartum period. In this retrospective observational study, we first investigated the presence of differing degrees of ketonuria (excluding acidosis) in women with GDM during labor. Second, we compared clinical characteristics among different degrees of ketonuria (Negative, Moderate, and Ketosis), as well as the duration of ketonuria and its distribution in different stages of labor. Finally, we analyzed adverse maternal and neonatal outcomes for each group.

## 2. Materials and Methods

We conducted a retrospective cohort study between April 2018 and April 2019 at the Department of Beijing Obstetrics and Gynecology Hospital, Capital Medical University. The cohort included only nulliparous women who had undergone singleton vaginal deliveries and had GDM. The inclusion criteria were ≥36 weeks of gestation in spontaneous labor. All patients exhibited good metabolic control during gestation and were eligible for vaginal delivery. The majority of patients exhibited diet-controlled GDM, whereas the remaining women required a small amount of insulin during pregnancy. Exclusion criteria were multiple pregnancies, multiparous women, and women with abnormal renal functions, including ketoacidosis, induced labor, precipitous delivery, hypertension, intrauterine growth restriction, chorioamnionitis, women delivering at <36 weeks of gestation, and no extant measurement of maternal ketonuria from the onset of labor.

The control of maternal blood sugar levels (BSLs) during labor and delivery conformed to the management recommended by the Joint British Diabetes Societies (JBDS) guidelines [16]. Once labor was established, we evaluated the maternal BSL hourly; and for all women with GDM on hourly monitoring, the BSL was maintained within a target range (4−7 mmol/L). VRIII was started if two consecutive BSL readings were above 7 mmol/L; 0.9% NaCl with 5% glucose was used as the substrate fluid with VRIII. The fluid was administered at 50 ml/h generally (the rate was adjusted to the volume status of the patient; see Supplemental Table cited from the JBDS guidelines for more details).

According to the manufacturer's protocol, ketonuria was classified into three levels: negative −/±, moderate +/++, and large +++/++++. The criteria for diagnosing ketosis were urinary ketones >++, and diagnosis of acidosis was a blood gas pH <7.3 and/or bicarbonate <15 mmol/L [14]. The ketonuria group was defined according to the highest ketonuria results during labor. According to the ketonuria level and diagnosis criteria for ketosis, women with GDM were classified into three groups: group 1 (Negative group), group 2 (Moderate group), and group 3 (Ketosis group).

Ketonuria was initially tested within 2 h of the onset of labor, and ketonuria was examined every 1-2 h during labor and delivery according to maternal BSL and ketonuria. When ketonuria was negative and maternal BSL was within the target range (4−7 mmol/L), ketonuria was examined every 2 h. When ketonuria was ≥+ or maternal BSL was above 7 mmol/L consecutively, ketonuria was examined hourly. As soon as ketonuria was ≥+, we routinely changed the ketonuria status by administering the food/drink, hydration, or IV 0.9% NaCl with 5% dextrose, as appropriate, if inadequate by mouth. If ketonuria status was still persistent, the affected women required an adjustment of VRIII (see Supplemental [Supplementary-material supplementary-material-1]). Arterial blood gas was analyzed if two consecutive ketosis levels were observed.

Background medical data for the pregnant woman and newborn were collected retrospectively from our antenatal care records and partograms including age, family history of diabetes, pregestational body mass index (BMI), and incidence of using insulin therapy during pregnancy. An oral glucose tolerance test (OGTT) and the homeostasis model assessment of insulin resistance (HOMA-IR) were recorded at 24−28 weeks of gestation. The serum levels of hemoglobin A1c (HbA1c), triglycerides (TG), cholesterol (TC), high-density lipoprotein (HDLc), and low-density lipoprotein (LDLc) were determined retrospectively at 34−36 weeks of gestation. Intrapartum maternal variables included gestational age at delivery, total duration of labor, BSL at the time of delivery, mode of delivery and incidence of epidural analgesia, use of oxytocin augmentation, fetal heart rate pattern III (FHR pattern III), meconium-stained amniotic fluid III (MSAF III), and postpartum hemorrhage. We analyzed the duration of the highest degree of ketonuria (from the onset of labor to delivery) according to different time periods (≤2 h, 3-4 h, 5-6 h, and >6 h). We also assessed the distribution of ketonuria at different stages of labor. Neonatal variables included birth weight, analysis of umbilical artery cord blood gas (pH), neonatal BSL at the time of delivery, incidence of low Apgar scores, and admission to the neonatal intensive care unit.

The criteria we used to determine the onset of labor were strong contractions at least every 5 minutes or an observed change in cervical dilation with an uncertain contraction pattern. Moreover, MSAF III was opaque and had thick meconium. The FHR pattern III was established according to the National Institute of Child Health and Human Development. Postpartum hemorrhage was defined as a bleeding volume of more than 500 mL during a vaginal delivery or more than 1000 mL during a cesarean section. The delivery neonatal BSL was that the one collected immediately prior to delivery. Neonatal hypoglycemia was defined as <2.6 mmol/L.

The statistical analysis was performed using the Statistical Package for the Social Sciences 19.0 (SPSS Inc., Chicago, IL, USA). In our study, continuous variables were all normally distributed and expressed as mean ± standard deviation (mean ± SD). Measurement data were compared using a one-way analysis of variance (ANOVA). If the ANOVA revealed a significant interaction between the variables, post hoc analyses were performed. Before the multiple comparisons were made, we looked at the significance of the variance homogeneity test. If it was more than 0.05, we carried out the Scheffe test to make multiple comparisons; otherwise, we chose the Tamhane's T2 test. In addition, categorical variables were expressed as percentages (%). The Pearson chi-square test and Fisher's exact test analysis were used for categorical variables. A *P* value of <0.05 was considered to be statistically significant.

## 3. Results

**Figure 1:**
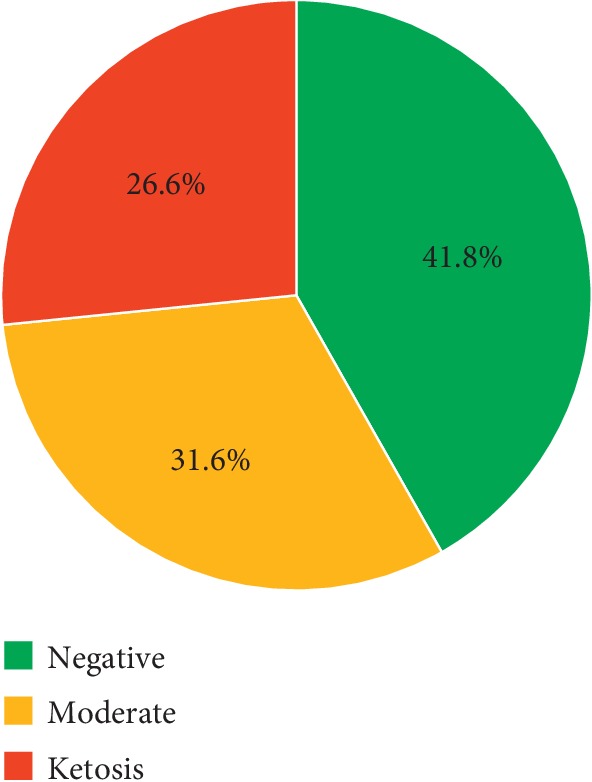
Study groups (KET classification).

A total of 570 deliveries by women with GDM were included in the study, and the differing degrees of ketonuria are summarized in Figure 1. Of the deliveries, 238 were negative for ketonuria (41.8%), 180 were moderate (31.6%), and 152 were ketosis (26.6%).

The associations between maternal baseline characteristics and ketonuria classification are summarized in Table 1.

The mean maternal age of the study group was 32.1 years (ranging from 20 to 42 years), and we did not find any relationship between maternal age and ketonuria during labor. Neither pregestational BMI and gestational gain weight nor rate of insulin use during pregnancy was different among the three groups. We did, however, observe statistically significant associations between ketonuria classification and a family history of diabetes (5.9% in the Negative group, 15.0% in the Moderate group, and 20.4% in the Ketosis group; *P* < 0.001). When we analyzed metabolic gestational characteristics, a significantly lower HOMA-IR was found in the Negative group (1.60 ± 0.74 in the Negative group, 2.14 ± 0.89 in the Moderate group, and 2.36 ± 1.11 in the Ketosis group; *P* < 0.001). In addition, the TG levels were significantly higher in the Ketosis group compared to the Negative and Moderate groups (3.32 ± 1.28 in the Ketosis group, 2.53 ± 0.90 in the Negative group, and 2.78 ± 1.20 in the Moderate group; *P* < 0.001). Moreover, the TC level significantly increased as the degree of ketonuria progressed (2.21 ± 0.70 in the Negative group, 3.60 ± 1.58 in the Moderate group, and 4.86 ± 1.52 in the Ketosis group; *P* < 0.001). Mean glucose at 0 h, 1 h, and 2 h and HbALc, HDLc, and LDLc levels were not significantly different among the three groups.

The intrapartum characteristics of the nulliparous women experiencing spontaneous labor are summarized in Table 2 according to ketonuria classification.

The mean gestational age of the study group was 39.1 ± 2.1 weeks (mean ± SD; range, 36–40 weeks), and there was no relationship between the ketonuria level and gestational age. Moreover, we did not uncover any relationship between birth weight or incidence of epidural analgesia and ketonuria level. There was, however, a significantly higher proportion of use of oxytocin augmentation in the Moderate and Ketosis groups compared to the Negative group (21.1% and 26.3% vs. 10.5%; *P* < 0.001). In addition, we observed a significantly lower maternal BSL at delivery in the Negative group, the majority of which fell within the target range of 4−7 mmol/L (6.67 ± 0.71 in the Negative group, 7.00 ± 0.47 in the Moderate group, and 7.13 ± 0.60 in the Ketosis group; *P* < 0.001). The level of neonatal BSL at delivery also significantly decreased as the degree of ketonuria progressed (4.27 ± 0.57 in the Negative group, 3.77 ± 0.58 in the Moderate group, and 3.56 ± 0.64 in the Ketosis group; *P* < 0.01). As to the total duration of labor, we observed a significantly longer duration of labor in the Ketosis group when compared to both the Negative and Moderate groups (13.85 ± 4.97 in the Ketosis group, 10.59 ± 4.88 in the Negative group, and 11.29 ± 4.83 in the Moderate group; *P* < 0.001). In addition, the Ketosis group had a significantly longer duration of ketonuria compared to the Moderate group (3.93 ± 1.54 vs. 2.87 ± 1.41, *P* < 0.001).

**Figure 2:**
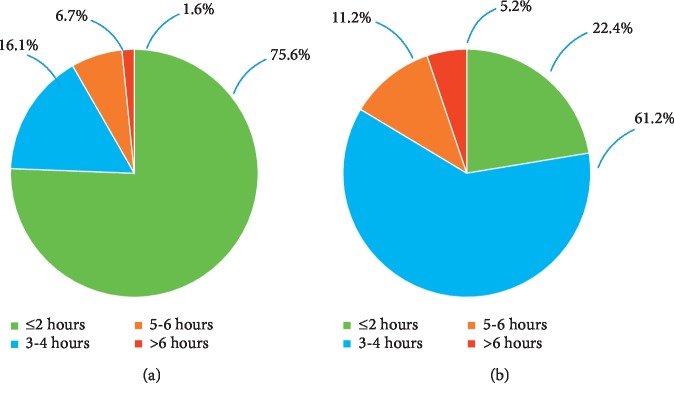
Proportion of different durations of ketonuria during labor in the (a) Moderate group and (b) Ketosis group.

**Figure 3:**
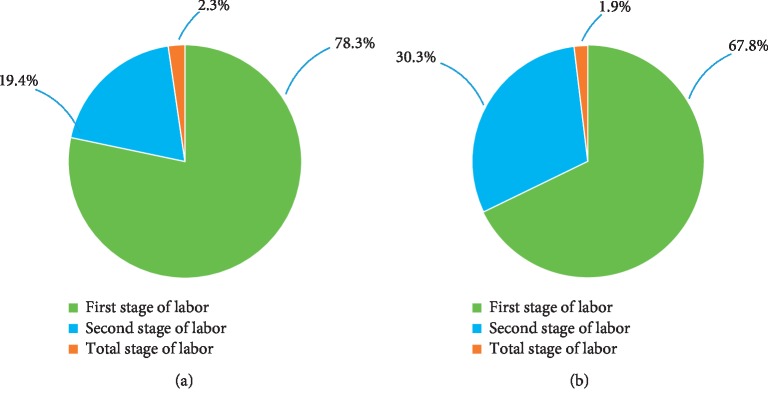
Proportion of distribution of ketonuria at different labor stages in the (a) Moderate group and (b) Ketosis group.

When we analyzed the different durations of ketonuria (Figure 2), we observed that in the Moderate group, 75.6% had a duration of moderate ketonuria of ≤2 h, 16.1% exhibited a duration of between 3 and 4 h, 6.7% exhibited a duration of 5-6 h, and 1.6% exhibited a duration of >6 h. In the Ketosis group, 22.4% of the duration of high ketonuria was ≤2 h, 61.2% exhibited a duration of 3-4 h, 11.2% exhibited a duration of 5-6 h, and 5.2% exhibited a duration of >6 h. The distribution of ketonuria at different stages of labor is shown in Figure 3; as the figure shows, most of the ketonuria state was resolved during the first stage (80% for the Moderate group and 68.4% for the Ketosis group).

The associations of maternal and neonatal outcomes with ketonuria classification are summarized in Table 3.

We observed a significantly greater incidence of FHR pattern III intrapartum as the degree of ketonuria progressed (0.9% in the Negative group, 7.2% in the Moderate group, and 14.5% in the Ketosis group; *P* < 0.001) and a significantly higher amount of MSAF III (4.6% in the Negative group, 11.7% in the Moderate group, and 17.8% in the Ketosis group; *P* < 0.001). In addition, a significantly higher number of cases with postpartum hemorrhage were found as ketonuria progressed (0.9% in the Negative group, 2.2% in the Moderate group, and 6.6% in the Ketosis group; *P*=0.004). Moreover, a significantly longer labor duration (>16 h) was observed in the Ketosis group compared to the Negative or Moderate groups (28.9% in the Ketosis group, 4.2% in the Negative group, and 9.4% in the Moderate group; *P* < 0.001). Regarding the mode of delivery, the vast majority (88.6%) were vaginal deliveries, and the rate of operative vaginal delivery significantly increased as ketonuria progressed 1.7% for the Negative group, 5.6% for the Moderate group, and 11.2% for the Ketosis group (*P* < 0.001). Of those women whose labor was converted to cesarean sections, the incidence in the Ketosis group increased 12.2 times compared with the Negative group and increased 1.8 times compared with the Moderate group (*P* < 0.001).

We also observed a significantly higher incidence of neonatal hypoglycemia in the Ketosis group compared with both the Negative and Moderate groups (9.9% in the Ketosis group, 3.4% in the Negative group, and 2.8% in the Moderate group; *P*=0.009). Moreover, we observed a significantly increased incidence of umbilical artery pH <7.2 as the degree of ketonuria increased (1.7% in the Negative group, 3.9% in the Moderate group, and 7.2% in the Ketosis group; *P*=0.022). Low Apgar scores (<7 at 1 min or 5 min) were rarely obtained in our study, and we observed no statistical difference in the degree of ketonuria. In addition, the number of neonatal intensive care admissions significantly increased as the degree of ketonuria progressed (4.2% in the Negative group, 7.8% in the Moderate group, and 12.5% in the Ketosis group; *P* < 0.001).

We further evaluated maternal and neonatal outcomes with respect to the duration of ketonuria and the distribution of ketonuria at different stages of labor in the Moderate group (summarized in Table 4).

We noted that the duration of ketonuria in the range of 3-4 h resulted in a significant proportion of cases being converted to cesarean sections (*P*=0.017). As the duration of moderate ketonuria gradually increased, there were also significant increases in operative vaginal deliveries, FHR pattern III, MSAF III, prolonged labor (>16 h), neonatal hypoglycemia, umbilical cord arterial pH <7.2, and neonatal intensive care admissions (*P* ≤ 0.001). When moderate ketonuria extended to the second stage of labor, we observed significantly increased operative vaginal deliveries, FHR pattern III, MSAF III, and prolonged labor (>16 h), compared to ketonuria that was limited to the first stage of labor (*P* < 0.01).

We also studied maternal and neonatal outcomes according to the different status durations of ketosis and the distribution of ketosis at different stages of labor in the Ketosis group (summarized in Table 5).

As the duration of ketosis gradually increased, there were significantly more cases with maternal and neonatal complications (*P* < 0.05), which was similar to the results observed in the Moderate group. With respect to ketosis distribution in the second stage of labor, cases with adverse outcomes were also the same as those observed in the Moderate group. Because cases with low Apgar scores (<7 at 1 min or 5 min) or with ketonuria persisting at all stages of labor were exceedingly rare in both the Moderate and Ketosis groups, we were unable to analyze these indices statistically.

## 4. Discussion

We demonstrated in our study that increased ketonuria is a common occurrence in labor. The high incidence of ketonuria in our study (31.6% for the Moderate group and 26.6% for Ketosis group) was consistent with that reported in other literature about ketonuria in the third trimester of pregnancy in women with GDM [8]. In our study, the majority of patients showed diet-controlled GDM, whereas the number of women requiring insulin during pregnancy was small. The rate of insulin use during pregnancy was not different among the three groups, which we attributed to good metabolic control during gestation or the small sample size.

As maternal BSL during labor was in the normal range in the majority of cases, the main causes of ketonuria in our study were reduced oral intake appropriate food and relative insulin resistance. Our study showed a significantly higher prevalence of a family history of diabetes complicated by ketonuria. Moreover, the levels of HOMA-IR during the second trimester were significantly higher in both ketonuria groups, which might have been caused by the anti-insulin characteristics exhibited by women with GDM [17]. However, the relationship between the levels of HOMA-IR in the third trimester and the degree of ketonuria in labor requires further study.

We also found that women with GDM complicated by ketonuria had higher plasma levels of cholesterol and triglycerides, especially in the Ketosis group. This phenomenon might be attributed to the ketone synthesis that takes place mainly in the liver in the mitochondrial matrices of hepatocytes and which is regulated by hormones. Hormones that stimulate lipolysis increase blood concentrations of free fatty acids (FFAs) (the starting materials for ketone body production) and enhance ketone formation [18]. Moreover, pregnant women tend to develop hyperlipidemia due to their elevated levels of estrogen. After a lengthy fast, hepatic glycogen stores are quickly depleted, and lipid mobilization is enhanced, resulting in ketone body generation. Therefore, gestational hypertriglyceridemia may enhance lipolysis and increase free fatty acids, thus boosting ketogenesis [19].

We analyzed the intrapartum characteristics of women with GDM who showed differing degrees of ketonuria and found a greater use of oxytocin augmentation and an increased duration of labor as the degree of ketonuria increased. This was consistent with other findings that ketone body accumulation inhibits uterine contractions and prolongs the duration of labor [20]. Adequate supplies of glucose are needed to maintain uterine contractions, which are important in the progress of labor; however, the metabolism of women with GDM complicated by ketonuria switches from utilizing glucose to using fat as its main fuel. In our study, maternal BSLs were higher in women with GDM complicated by ketonuria, which might have been due to their relative insulin deficiency or insulin resistance. Correspondingly, neonatal BSL at the time of delivery significantly decreased as the degree of ketonuria progressed, which might have been related to the increased maternal BSL at delivery. Our results were consistent with previous studies reviewed by NICE, which suggested that maternal hyperglycemia during labor is associated with an increased risk of neonatal hypoglycemia [14]. We also demonstrated that the Ketosis group exhibited a significantly longer duration of ketonuria compared to the Moderate group, suggesting that the ketosis state is linked to severe dehydration and severe insulin resistance, which were both more difficult to resolve relative to the group with moderate ketonuria.

Our study depicted the effects of ketonuria accompanied by increased maternal postpartum hemorrhage, the need for forceps-assisted delivery, and augmented conversion to cesarean sections. In addition to prolonged labor and inadequate uterine contractions, subsequent fetal distress followed by MSAF III and an abnormal FHR pattern III could also explain the increased rate of operative vaginal deliveries and cesarean sections. In our study, the main indicator of an operative vaginal delivery was fetal distress; for conversion to a cesarean section, the main indicator was relative cephalopelvic monosymmetry.

In addition, we demonstrated a more pathologic FHR pattern III intrapartum as maternal ketonuria progressed, which was consistent with a previous study showing that abnormal fetal heart physiology in the setting of postterm pregnancy was related to maternal ketonuria [6]. Additional support for our results comes from recent studies reporting pathologic fetal heart tracings in the presence of extreme quantities of ketone bodies, such as those in diabetic ketoacidosis (DKA), starvation ketoacidosis (SKA), and euglycemic diabetic ketoacidosis (EDKA) [21, 22]. Our data also depicted an increased incidence of FHR pattern III as the durations of both moderate ketonuria and ketosis were prolonged, especially when the duration of ketosis was >4 h. Based on our results, we recommend enhanced FHR monitoring during the second stage of labor in pregnant women with both moderate ketonuria and ketosis.

We hypothesized that the presence of increased FHR pattern III is not only due to maternal dehydration from exposure to ketone bodies but also due to neurologic alterations in some fetuses exposed to maternal ketones. Support for our hypothesis is provided by the documented impact ketonemia has on CNS excitability in adult offspring as well as on the fetus [9]. In addition, several theories have attempted to explain the mechanisms underlying the impact of ketones on CNS functioning. According to one of these theories, ketone metabolism causes reduced activity of the ATP-dependent pump in membrane channels (with the pump contributing to the stabilization of cellular membranes), and this can affect neuronal activity [23]. Another theory, noted in adult mouse offspring, is that ketones stimulate gamma aminobutyric acid (GABA) synthesis, which is an inhibitory neurotransmitter of brain excitability [24]. Animal experiments on the fetuses of ketogenic mothers have shown embryonic growth abnormalities and a reduced volume of cerebral cortex, hippocampus, corpus callosum, and lateral brain ventricles [25].

In the current study, we also demonstrated that the presence of MSAF III was associated with an increased degree of maternal ketonuria intrapartum. Meconium release in the intrapartum period is related either to fetal maturity or hypoxia [26], and while primary meconium is related more to fetal maturation, secondary meconium more likely reflects intrapartum fetal distress [27]. Interestingly, all the amniotic fluid we detected ranged from clear to meconium (secondary meconium). We hypothesized that maternal ketones may change fetal homeostasis and affect brain excitability as mentioned above. It is possible that the stimulation of the vagus nerve during high ketone production or hypoxia may cause peristalsis of the bowel and relaxation of the anal sphincter, leading to the passage of meconium into the amniotic cavity. However, whether maternal ketones directly affect the fetal vagus nerve deserves further study.

We also observed a significantly increased rate of neonatal complications as the degree of ketonuria increased, including neonatal hypoglycemia, low umbilical cord arterial pH, low Apgar scores, and increased neonatal intensive care admissions. This was especially true when the duration of ketosis was >4 h and ketosis extended to the second stage of labor. We rarely observed low Apgar scores in our study, but this might have been due to our strict ketone monitoring and elimination. Our primary indicator for neonatal intensive care admission was respiratory distress.

The adverse neonatal outcomes we uncovered may be related to ketone bodies participating in maternal metabolic derangements and even changing fetal homeostasis. Long-standing ketonuria is considered to be a surrogate measure of both dehydration and caloric balance [28], and maternal ketonemia that results in ketonuria is a hyperosmolar condition [29]. Studies on sheep showed that maternal conditions involving hyperosmolarity (such as maternal dehydration) resulted in a diminution of fetal amniotic fluid volume from decreased fetal urination [30], potentially due to attenuated fetal production of atrial natriuretic factor [31]. Similarly, several human studies showed that maternal hydration resulted in a significant increase in amniotic fluid volume [32]. Moreover, investigators have detected within 5 min in fetal plasma the presence of radio-labeled *β*-hydroxybutyrate (one of three ketone bodies) administered to pregnant rats through the femoral vein [33]. Other studies of offspring exposed in utero to maternal dehydration showed evidence of specific nutritional deficiencies affecting fetal brain development [34].

There were a few limitations to our study. First, it was a retrospective design. Second, because this study was performed at a single center and included a small cohort size, the small number of low Apgar scores could not be analyzed statistically among women with GDM with differing durations and distributions of ketonuria. Moreover, the few adverse maternal and neonatal outcomes could not be appropriately analyzed with correlation and regression analyses. Finally, maternal ketonuria is considered an indirect cause of changes in fetal homeostasis. We did not assess the relationship between maternal ketonemia and fetal ketonemia at delivery, so this needs to be further addressed. However, our study was the first to emphasize the importance of surveying the degrees of ketonuria and the duration and distribution of ketonuria in nulliparous women with GDM during labor.

## 5. Conclusions

In conclusion, we showed in the present study that maternal ketonuria is fairly common in the intrapartum period for nulliparous women with GDM. When ketonuria manifests, the fetus may face increased risks of maternal and neonatal complications and increased operative vaginal deliveries and conversions to cesarean sections. Therefore, we suggest that in the context of ketonuria, monitoring of the fetal heart rate during labor and delivery should be increased in both moderate ketonuria and ketosis states. That being said, our results need to be further confirmed by prospective studies and the use of regression analyses. Eventually, the presence of ketonuria during labor will require intensive monitoring, and randomized studies should be undertaken to determine the effects of treating maternal ketonuria on perinatal outcomes.

## Figures and Tables

**Table 1:** Baseline characteristics by ketonuria classification of nulliparous women with spontaneous labor.

Characteristics	NG (−/±)	MG (+/++)	KG (+++/++++)	Overall *P* value	Multiple comparison
Age (years)	31.84 ± 3.34	32.44 ± 3.23	32.24 ± 3.19	0.154	—
Pregestational BM (kg/m^2^)	22.97 ± 3.33	22.96 ± 3.22	23.28 ± 3.58	0.616	—
Gain weight (kg)	13.87 ± 2.86	13.59 ± 2.73	13.80 ± 2.86	0.539	—
Family history of diabetes, *n* (%)	14 (5.9)	27 (15.0)	31 (20.4)	<0.001	—
Using insulin therapy during pregnancy, *n* (%)	6 (2.5)	11 (6.1)	9 (5.9)	0.147	—
Pregnancy metabolic indicator					
Glucose 0 h (OGTT) (mmol/l)	4.91 ± 0.40	4.88 ± 0.44	4.92 ± 0.46	0.673	—
Glucose 1 h (OGTT) (mmol/l)	9.59 ± 1.50	9.87 ± 1.30	9.92 ± 1.30	0.064	—
Glucose 2 h (OGTT) (mmol/l)	7.76 ± 1.34	8.07 ± 1.37	8.07 ± 1.30	0.075	—
HbALc (%)	5.16 ± 0.35	5.23 ± 0.30	5.17 ± 0.29	0.066	—
HOMA-IR (IU *∗* mmol/L)	1.60 ± 0.74	2.14 ± 0.89	2.36 ± 1.11	<0.001	NG < MG, TG
TG (mmol/L)	2.53 ± 0.89	2.78 ± 1.19	3.32 ± 1.28	<0.001	NG, MG < TG
TC (mmol/L)	2.21 ± 0.70	3.60 ± 1.58	4.86 ± 1.52	<0.001	NG < MG < TG
HDLc (mmol/L)	1.63 ± 0.31	1.71 ± 0.36	1.70 ± 0.33	0.055	—
LDLc (mg/dl)	2.90 ± 0.73	2.89 ± 0.79	2.91 ± 0.70	0.960	—

Data are presented as mean ± SD or *n* (%). Overall *P* values for continuous variables determined using ANOVA and for categorical variables determined using chi-square or Fisher's exact tests. Multiple comparisons among the three groups were calculated using post hoc analysis (Scheffe or Tamhane's T2 test). NG: negative group; MG: moderate group; TG: ketosis group.

**Table 2:** Intrapartum characteristics of nulliparous women with spontaneous labor by ketonuria classification.

Characteristics	NG (−/±)	MG (+/++)	KG (+++/++++)	Overall *P* value	Multiple comparison
Gestational age (weeks)	38.7 ± 1.2	38.9 ± 1.8	38.9 ± 0.9	0.277	—
Birth weight (grams)	3370 ± 396.2	3408 ± 388.5	3413 ± 359.9	0.460	—
Epidural analgesia, *n* (%)	182 (76.5)	137 (76.1)	116 (76.3)	0.997	—
Oxytocin use during labor, *n* (%)	25 (10.5)	38 (21.1)	40 (26.3)	<0.001	—
Maternal BSL at delivery (mmol/L)	6.67 ± 0.71	7.00 ± 0.47	7.13 ± 0.60	<0.001	NG < MG, TG
Neonatal BSL at delivery (mmol/L)	4.27 ± 0.57	3.77 ± 0.58	3.56 ± 0.64	<0.001	NG < MG < TG
Duration of labor (hours)	10.59 ± 4.88	11.29 ± 4.83	13.85 ± 4.97	<0.001	NG, MG < TG
Umbilical cord arterial pH	7.33 ± 0.06	7.31 ± 0.08	7.30 ± 0.73	0.008	NG < TG
Duration of ketonuria (hours)	—	2.87 ± 1.41	3.93 ± 1.54	<0.001	MG < TG

Data are presented as mean ± SD or *n* (%). Overall *P* values for continuous variables determined using ANOVA and for categorical variables determined using chi-square or Fisher's exact tests. Multiple comparisons among the three groups were calculated using post hoc analysis (Scheffe or Tamhane's T2 test). NG: negative group; MG: moderate group; TG: ketosis group.

**Table 3:** Maternal and neonatal outcomes by ketonuria classification of nulliparous women with spontaneous labor.

Characteristics	NG (−/±)	MG (+/++)	KG (+++/++++)	*P* value
Operative vaginal delivery, *n* (%)	4 (1.7)	10 (5.6)	17 (11.2)	<0.001
Conversion to cesarean section, *n* (%)	5 (1.3)	16 (8.9)	24 (15.8)	<0.001
FHR intrapartum pattern III, *n* (%)	2 (0.9)	13 (7.2)	22 (14.5)	<0.001
MSAF III, *n* (%)	11 (4.6)	21 (11.7)	27 (17.8)	<0.001
Prolonged labor >16 hours, *n* (%)	10 (4.2)	17 (9.4)	44 (28.9)	<0.001
Postpartum hemorrhage, *n* (%)	2 (0.9)	4 (2.2)	10 (6.6)	0.004
Neonatal hypoglycemia, *n* (%)	8 (3.4)	5 (2.8)	15 (9.9)	0.009
Umbilical cord arterial pH <7.2, *n* (%)	4 (1.7)	7 (3.9)	11 (7.2)	0.022
Adverse neonatal outcomes, *n* (%)				
Apgar score <7				
1 min	0 (0)	2 (0)	3 (1.06)	0.067
5 min	0 (0)	1 (0.24)	2 (0.71)	0.117
Neonatal care unit admission, *n* (%)	10 (4.2)	14 (7.8)	19 (12.5)	<0.001

Data are presented as *n* (%). Categorical variables determined using chi-square or Fisher's exact tests. NG: negative group; MG: moderate group; TG: ketosis group.

**Table 4:** Maternal and neonatal outcomes of GDM women with spontaneous labor in the Moderate group.

Characteristics	Duration of ketonuria					Distribution of ketonuria during labor		
	≤2 hours	3-4 hours	5-6 hours	>6 hours	*P* value	First stage of labor	Second stage of labor	*P* value
Operative vaginal delivery, *n* (%)	1 (0.7)	4 (13.8)	3 (25.0)	2 (66.7)	<0.001	3 (2.1)	7 (20.0)	0.001
Conversion to cesarean section, *n* (%)	0 (0)	14 (48.3)	2 (16.7)	0 (0)	=0.017	16 (11.1)	0 (0)	—
FHR intrapartum pattern III, *n* (%)	2 (1.5)	4 (13.8)	5 (41.7)	2 (66.7)	<0.001	3 (2.1)	9 (25.7)	<0.001
MSAF III, *n* (%)	5 (3.7)	7 (24.1)	7 (58.3)	2 (66.7)	<0.001	5 (3.5)	16 (45.7)	<0.001
Postpartum hemorrhage, *n* (%)	0 (0)	1 (3.5)	2 (16.7)	1 (33.3)	=0.001	2 (1.4)	2 (5.7)	0.177
Prolonged labor >16 hours, *n* (%)	5 (3.7)	6 (20.7)	4 (33.3)	2 (66.7)	<0.001	8 (5.7)	8 (22.9)	0.004
Neonatal hypoglycemia, *n* (%)	0 (0)	2 (6.9)	2 (16.7)	1 (33.3)	<0.001	2 (1.4)	2 (5.7)	0.177
Umbilical cord arterial pH <7.2, *n* (%)	0 (0)	2 (6.9)	3 (25.0)	2 (66.7)	<0.001	2 (1.4)	3 (8.6)	0.054
Neonatal care unit admission, *n* (%)	2 (1.5)	5 (17.2)	5 (41.7)	2 (66.7)	<0.001	8 (5.7)	5 (14.3)	0.089

Data are presented as *n* (%). Categorical variables determined using chi-square or Fisher's exact tests.

**Table 5:** Maternal and neonatal outcomes of nulliparous women with spontaneous labor in Ketosis group.

Characteristics	Duration of ketosis					Distribution of ketosis during labor		
	≤2 hours	3-4 hours	5-6 hours	>6 hours	*P* value	First stage of labor	Second stage of labor	*P* value
Operative vaginal delivery, *n* (%)	0 (0)	7 (7.5)	6 (35.3)	4 (50.0)	<0.001	5 (4.9)	11 (23.9)	=0.001
Conversion to cesarean section, *n* (%)	4 (11.8)	8 (8.6)	5 (29.4)	7 (87.5)	<0.001	24 (23.3)	0 (0)	—
FHR intrapartum pattern III, *n* (%)	5 (14.7)	8 (23.3)	4 (23.5)	5 (62.5)	=0.001	10 (9.7)	11 (23.9)	<0.001
MSAF III, *n* (%)	5 (14.7)	11 (11.8)	5 (29.4)	6 (75.0)	<0.001	5 (4.9)	21 (45.7)	<0.001
Postpartum hemorrhage, *n* (%)	0 (0)	5 (5.4)	4 (23.5)	1 (12.5)	=0.014	7 (6.8)	3 (6.5)	=1.000
Prolonged labor >16 hours, *n* (%)	6 (17.6)	21 (22.6)	13 (76.5)	4 (50.0)	<0.001	32 (31.1)	12 (26.1)	=0.538
Neonatal hypoglycemia, *n* (%)	0 (0)	4 (4.3)	6 (35.3)	5 (62.5)	<0.001	4 (3.9)	10 (21.7)	=0.001
Umbilical cord arterial pH <7.2, *n* (%)	1 (2.9)	3 (3.2)	3 (17.6)	4 (50.0)	<0.001	4 (3.9)	5 (10.9)	=0.135
Neonatal care unit admission, *n* (%)	2 (5.9)	9 (9.7)	5 (29.4)	3 (37.5)	=0.014	9 (8.7)	5 (10.9)	=0.680

Data are presented as *n* (%). Categorical variables determined using chi-square or Fisher's exact tests.

## Data Availability

The data used to support the findings of this study are available from the corresponding author upon request.

## Supplementary Materials

Supplementary Materials Our supplementary material file was about the protocol of intravenous insulin for labor in our study. Supplemental Table: intravenous insulin prescription and protocol for labor.
