# Supplementary material for: Intrapartum Results on Differing Degrees of Ketonuria in Nulliparous Women with Gestational Diabetes Mellitus during Spontaneous Labor
Source: Int J Endocrinol. 2019 Nov 11;2019:7207012. doi: 10.1155/2019/7207012 (PMC6885219; doi:10.1155/2019/7207012)
Supplement: Supplementary Materials — Our supplementary material file was about the protocol of intravenous insulin for labor in our study. Supplemental Table: intravenous insulin prescription and protocol for labor. [file 7207012.f1.docx]

| **Supplemental Table Intravenous insulin prescription and protocol for labor** | | | |
| --- | --- | --- | --- |
|  | **DOSING ALGORITHM**  **（Please see the guide below)** | | |
| **Algorithm** | 1 | 2 | 3 |
|  | For most women | For women not controlled on algorithm 1 or needing >80units/day of insulin | For women not controlled on algorithm 2 |
| **BSL Levels (mmol/L)** | Infusion Rate (units/hr=ml/hr) | | |
| <4 | Stop insulin for 20 minutes  Treat hypo as per guideline (re-check BSL in 10minutes) | | |
| 4.0-5.5 | 0.2 | 0.5 | 1.0 |
| 5.6-7.0 | 0.5 | 1.0 | 2.0 |
| 7.1-8.5 | 1.0 | 1.5 | 3.0 |
| 8.6-11.0 | 1.5 | 2.0 | 4.0 |
| 11.1-14.0 | 2.0 | 2.5 | 5.0 |
| 14.1-17.0 | 2.5 | 3.0 | 6.0 |
| 17.1-20.0 | 3.0 | 4.0 | 7.0 |
| >20.1 | 4.0 | 6.0 | 8.0 |
